# The affordance-matching hypothesis: how objects guide action understanding and prediction

**DOI:** 10.3389/fnhum.2014.00254

**Published:** 2014-05-12

**Authors:** Patric Bach, Toby Nicholson, Matthew Hudson

**Affiliations:** School of Psychology, University of Plymouth, Drake CircusDevon, UK

**Keywords:** affordances, action understanding, action prediction, object function, object manipulation

## Abstract

Action understanding lies at the heart of social interaction. Prior research has often conceptualized this capacity in terms of a motoric matching of observed actions to an action in one’s motor repertoire, but has ignored the role of object information. In this manuscript, we set out an alternative conception of intention understanding, which places the role of objects as central to our observation and comprehension of the actions of others. We outline the current understanding of the interconnectedness of action and object knowledge, demonstrating how both rely heavily on the other. We then propose a novel framework, the affordance-matching hypothesis, which incorporates these findings into a simple model of action understanding, in which object knowledge—what an object is for and how it is used—can inform and constrain both action interpretation and prediction. We will review recent empirical evidence that supports such an object-based view of action understanding and we relate the affordance matching hypothesis to recent proposals that have re-conceptualized the role of mirror neurons in action understanding.

## Action understanding in an object context: the affordance-matching hypothesis

Action understanding lies at the heart of social interaction. Knowing the goal of another person’s action allows one to infer their internal states, predict what they are going to do next, and to coordinate one’s own actions with theirs (Hamilton, 2009; Sebanz and Knoblich, 2009; Bach et al., 2011). The ability to understand others’ actions is often assumed to rely on specialized brain systems that “directly map” observed motor acts to a corresponding action in the observer’s motor repertoire, allowing it to be identified and its goal to be derived (Rizzolatti et al., 2001; Gazzola and Keysers, 2009). In monkeys, mirror neurons have been discovered that fire both when the monkey executes a particular action, and when it merely observes the same actions being executed by someone else (Pellegrino et al., 1992; Gallese et al., 1996). Also for humans, there is now converging evidence that action observation engages neuronal ensembles also involved in action execution, and that these ensembles code specific actions across both domains (Fadiga et al., 1995; Chong et al., 2008; Mukamel et al., 2010; Oosterhof et al., 2010, 2012).

Yet, even though there remains little doubt that action-related representations are also activated when one observes others act, attempts to directly link these activations to goal understanding have been less successful. There is little evidence from lesion or transcranial magnetic stimulation studies that would reveal a critical role of motor-related brain areas for understanding the actions of others (Catmur et al., 2007; Negri et al., 2007; Kalénine et al., 2010; but see Avenanti et al., 2013b; Rogalsky et al., 2013). Similarly, whereas some imaging studies revealed an involvement of mirror-related areas in action understanding tasks, such as the inferior frontal gyrus or the anterior intraparietal sulcus (Iacoboni et al., 2005; Hamilton and Grafton, 2006), a growing number of studies point to areas outside the classical observation-execution matching system, such as the medial prefrontal cortex, the superior temporal sulcus, or the posterior temporal lobe (Brass et al., 2007; de Lange et al., 2008; Liepelt et al., 2008b; Kalénine et al., 2010). Others reveal that mirror-related brain activations are primarily found for meaningless actions, where kinematics is the only information available (Hétu et al., 2011), substantially limiting the theoretical reach of motoric matching accounts. Finally, there are theoretical reasons why motor or kinesthetic information, on which direct matching is assumed to be based, does not suffice to unambiguously identify the goals of complex human motor acts. For example, most human motor behaviors (e.g., picking up something) can be performed in various circumstances to achieve a variety of goals, such that a one-to-one mapping of actions to goals is not possible (e.g., Hurford, 2004; Jacob and Jeannerod, 2005; Uithol et al., 2011).

These observations have posed a challenge to motor-matching views of action understanding, and have led several theorists to suggest either that the direct-matching account has to be revised, or that motoric matching cannot be the primary driver of action understanding in humans (Bach et al., 2005, 2011; Csibra, 2008; Kilner, 2011). Here, we propose a new view, which incorporates the available data on motoric matching and mirror neurons, but places them in a model of action understanding that emphasizes the role of object knowledge, which helps predict and interpret any observed motor act. Such a combined model, we argue, can explain extant data and account for several of the observed inconsistencies. In the following, we will (1) briefly review the current understanding of action knowledge associated with objects; (2) sketch a basic model of how this knowledge could contribute to action understanding, and (3) review common findings in humans and monkeys on the use of object-related knowledge in action observation in the light of this model.

Throughout the manuscript we use the term “goal” to refer to desired states of the environment, one’s own body, or mind. Following Csibra (2008), we presuppose that goals can be located at different levels, reaching from simple, low level goals, such as completing a grasp or hammering in a nail, to distal goals such as hanging up a picture frame. We use the term “action” to refer to bodily movements that are performed with the express purpose to achieve such a goal. The term “target objects” or “recipient objects” are used to refer to the objects affected by these actions.

## Action information provided by objects

The effective use of objects sets humans apart from even their closest relatives in the animal kingdom (e.g., Johnson-Frey, 2003). Most human actions involve objects, either as the recipient to be *acted upon*, or as a tool to be *acted with* (cf. Johnson-Frey et al., 2003). The capacity to use objects has unlocked a vast range of effects humans can achieve in the environment that would otherwise be outside the scope of their effector systems. They range from cutting with a knife, shooting a gun, to sending a text message with a mobile phone, and traveling the world with various types of vehicle.

The capacity for using these objects is underpinned by a specialized network in the left hemisphere, spanning frontal, parietal and temporal regions (Haaland et al., 2000; Johnson-Frey, 2004, for review; Binkofski and Buxbaum, 2013; for reviews, see van Elk et al., 2013), some of which appear to be unique to humans (Orban et al., 2006; Peeters et al., 2009, 2013). This network supports object-directed action by coding (at least) two types of information. For every object, humans learn not only what goals they can, in principle, achieve with it (“function knowledge”), but also the motor behaviors that are required to achieve these goals (“manipulation knowledge”) (Kelemen, 1999; Buxbaum et al., 2000; Buxbaum and Saffran, 2002; Casby, 2003, for a review, see van Elk et al., 2013). When growing up, one learns, for example, that a tap is for getting water, and that this requires turning it clockwise. Similarly, one learns that a knife is for cutting, and that this requires alternating forward and backwards movements, with an amount of downward pressure that depends on the object one wants to cut. Objects, therefore, seem to provide one with the same links between potential action outcomes and required motor behaviors that are central to the control of voluntary action (see Hommel et al., 2001). These links allow objects to act as an interface between an actor’s goals and their motor system (cf. van Elk et al., 2013). They allow actors not only to decide *whether* they want to use an object (by matching object functions to one’s current goals), but also—if they do—to derive *how* to utilize the object to achieve the desired result (by using manipulation knowledge to guide one’s motor behaviors with the object).

Whenever people interact with objects at least some aspects of this knowledge are activated automatically (e.g., Bub et al., 2003, 2008). In the monkey premotor cortex, so called *canonical* *neurons* have been discovered that fire not only when the monkey executes a specific grip (e.g., a precision grip), but also if it merely observes an object which requires such a grip (a small object such as a peanut), indicating a role in linking objects to actions (Murata et al., 1997). Similar evidence comes from behavioral and imaging studies in humans. Passively viewing an object, for example, has been shown to activate not only the basic movements for reaching and grasping it (e.g., Tucker and Ellis, 1998, 2001; Grèzes et al., 2003; Buccino et al., 2009), but also—under appropriate circumstances—the more idiosyncratic movements required for realizing the objects’ specific functions (e.g., the swinging movement required to hammer in a nail; for a review, Creem and Proffitt, 2001; Bach et al., 2005; Bub et al., 2008; van Elk et al., 2009; see van Elk et al., 2013).

Action information is such a central aspect of human object knowledge that it directly affects object identification and categorization. Already in 12 month old infants, object function contributes to object individuation and categorization (e.g., Booth and Waxman, 2002; Kingo and Krøjgaard, 2012). In adults, several studies have shown that an object is identified more easily when preceded by an object with either a similar or complementary function (e.g., corkscrew, wine bottle) (e.g., Riddoch et al., 2003; Bach et al., 2005; McNair and Harris, 2013), or one that requires similar forms of manipulation (e.g., both a piano and a keyboard require typing, Helbig et al., 2006; McNair and Harris, 2012). These results are mirrored on a neurophysiological level by fMRI repetition suppression effects for objects associated with similar actions, even when these objects are only passively viewed (e.g., Yee et al., 2010; Valyear et al., 2012).

Other studies document the tight coupling of function and manipulation knowledge (see van Elk et al., 2013 for a review). Several imaging studies have revealed at least partially overlapping cortical representations for function and manipulation knowledge (Kellenbach et al., 2003; Boronat et al., 2005; Canessa et al., 2008). Similarly, it has been known for a long time that lesions to the left-hemispheric tool networks disrupt knowledge not only of what the objects are “for”—goals that can achieved with them—but also knowledge of how they have to be used, while disruptions of function knowledge only are rare (Ochipa et al., 1989; Hodges et al., 1999; Haaland et al., 2000; Buxbaum and Saffran, 2002; Goldenberg and Spatt, 2009). In addition, there is a host of behavioral studies demonstrating that the activation of manipulation knowledge is tied to the prior activation of function/goal information, both on the behavioral (Bach et al., 2005; van Elk et al., 2009; McNair and Harris, 2013) and on the neurophysiological level (Bach et al., 2010b). For example, in a recent study based on Tucker and Ellis (1998) classic affordance paradigm, it was shown that which of an object’s manipulation was retrieved—grasping for placing or for functional object use—was determined by which goal was suggested by the surrounding context (see also Valyear et al., 2011; Kalénine et al., 2013).

## The affordance-matching hypothesis

The basic assumption of the affordance-matching hypothesis is that manipulation and function knowledge about objects cannot only be used during action execution, but also for predicting and understanding the actions of others. In the same way as object function and manipulation knowledge can act as the interface between one’s own goal and motor systems, it can provide one with similar links between the inferred goals of others and their likely motor behaviors.

**Figure 1:**
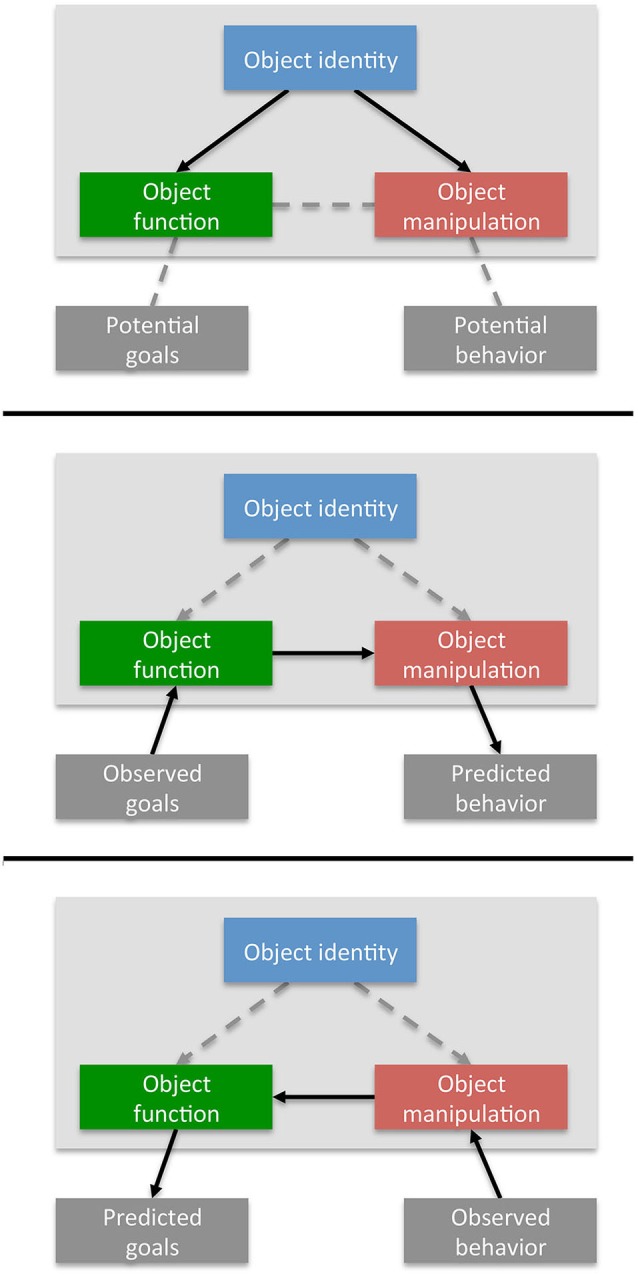
**Affordance matching during action observation**. Top panel: object identification provides information about what an object is for (function knowledge) and how it has to be manipulated to realize this function (manipulation knowledge). Middle panel: flow of information during action prediction. Inferred goals of an actor activates objects with matching functions. The associated manipulation knowledge predicts forthcoming movements. Bottom panel: flow of information during action interpretation. Observed behavior that matches an object’s manipulation activates the corresponding function, which in turn provides information about the actor’s goal.

The affordance-matching hypothesis has two main features. The first feature is the assumption that whenever we see somebody else in the vicinity of objects, the associated function and manipulation knowledge is retrieved (see Figure 1, top panel, cf. Rochat, 1995; Stoffregen et al., 1999; Costantini et al., 2011; Cardellicchio et al., 2013; for a review, see Creem-Regehr et al., 2013), constrained by further contextual cues such as other objects or social signals (see below). As is the case for one’s own actions, this provides the observer with immediate knowledge about the potential goals of the actor (through function knowledge: what the objects are *for)*, as well as the bodily movements that would be required to achieve these goals (through manipulation knowledge: *how* the objects have to be used). Imagine, for example, the unpleasant situation of standing across from another person holding a gun. Object knowledge specifies that a gun is for shooting (function knowledge), and that, in order to achieve this goal, the gun would have to be raised, pointed at the target, and fired (manipulation knowledge). Thus, simply deriving function and manipulation knowledge about the objects somebody acts with—without taking into account the specific motor behavior they perform—can serve both interpretative and predictive roles. Function knowledge supports *action interpretation* because knowledge about what an object is for provides insights into the potential goals of the other person. In contrast, manipulation knowledge aids *action prediction*, because knowledge about how an object is handled highlights potentially forthcoming actions, supporting more efficient identification and interaction.

The second major feature of the affordance-matching hypothesis is the assumption that, as during action production, an object’s function and manipulation knowledge are coupled, so that activating one also activates the other. This coupling substantially enhances the predictive and interpretative contributions of object knowledge, depending on the flow of information for function to manipulation knowledge or vice versa (Figure 1, middle and lower panel). Consider, for example, that most objects have multiple uses—even the gun could be given to someone, holstered, or harmlessly laid on a table—and there are typically multiple objects in a scene, each associated with a number of functional manipulations. We assume that these objects are not weighted equally during action observation. Instead, as it is the case during own action planning (e.g., Valyear et al., 2011; Kalénine et al., 2013), those objects will be highlighted, the functions of which are most in line with the (inferred) goals of the actor. Moreover, because object knowledge ties these functions to specific manipulations, the identification of such a functionally matching object can directly activate the associated motor behaviors, leading to action predictions that are in line with the inferred goals (Figure 1, middle panel).

**Figure 2:**
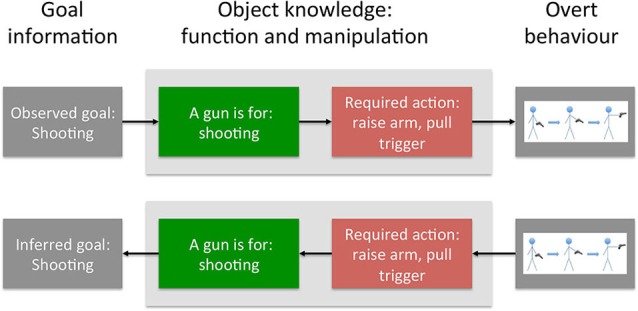
**Concrete example for the flow of information during action prediction and interpretation**. Top panel: Action prediction. Prior knowledge of an actor’s goal (shooting) activates knowledge of objects with corresponding function. The associated manipulation knowledge (raising the arm, pulling the trigger) supports action prediction by biasing visual perception towards these manipulations. Lower panel: Action interpretation. Observed behavior is matched to the manipulations supported by the object. If both match, the corresponding functions are activated, providing likely goals of the actor.

Previous research has established that additional objects in the environment—especially potential recipients of the action—are another major determinant for which action goals are pre-activated. Seeing a person holding a hammer might activate hammering movements to a stronger extent when this person is also holding a nail than when they are holding a toolbox (cf. Bach et al., 2005, 2009, 2010b; Yoon et al., 2012; McNair and Harris, 2013). Social cues are another important influence, as cues such as gaze or emotional expression can directly supply action goals. In the above example, if the person shows an angry facial expression and tone of voice, his actions of raising the arm and pulling the trigger will be foremost in our mind (Figure 2; upper panel), while a calm voice and friendly manner might at least make us consider the other possible meaningful actions one can do with a gun.

Here, therefore, flow of information from object function to manipulation aided action prediction. In contrast, the interpretation of observed motor behavior can benefit from the reverse flow of information: from manipulation to function knowledge. Note that, in many cases, an observed motor act is, by itself, devoid of meaning. The same—or at least very similar—motor act can be used for various purposes. Consider the everyday actions of inserting a credit card into a cash machine, or a train ticket into a ticker canceller. Motorically, both actions are virtually identical, but they serve very different goals (cf. Bach et al., 2005, 2009, 2010b; Jacob and Jeannerod, 2005). However, knowledge about the objects involved can directly disambiguate such alternative interpretations. Because object knowledge links the different manipulations of a tool with distinct functions, the detection of a motor behavior that matches such a manipulation can directly confirm the associated action goal (Figure 1, lower panel). In the above example, if the person with the gun in the hand indeed raises their arm, the interpretation is clear: with a gun in the hand, the otherwise meaningless motion of raising the arm is predicted by the goal of shooting (Figure 2, lower panel).

This interpretative role of object knowledge becomes particularly important if one considers that not only motor acts are ambiguous, but the functions of objects are as well. Some objects can be handled in different ways, and produce different outcomes. For example, a fork can be used to spear a carrot (in order to subsequently eat it) or to mash it. Here, the object context is identical and therefore does not allow one to anticipate one of these goals. However, a match of the actually observed motor behavior with one of the objects’ functional uses immediately provides such disambiguating information. As a consequence, just seeing how the fork is held may be enough to disambiguate its subsequent use.

Together, therefore, the affordance-matching hypothesis specifies the different pathways of how objects—via the associated function and manipulation knowledge—can make powerful contributions to both action interpretation and action prediction. For descriptive purposes, the flow of information through these pathways has been described mostly separately. Of course, interpretation and prediction in most cases interact strongly, with one constantly influencing the other. For example, a confirmed action prediction will verify inferred action goals, which, in turn, will trigger new action predictions, that can be either confirmed or disconfirmed by new sensory evidence.

## Evidence for affordance matching in action observation

Several recent studies have documented the major role of object information in action understanding (e.g., Hernik and Csibra, 2009; Hunnius and Bekkering, 2010; Bach et al., 2014). They do not only show that object-based modes of action understanding can complement the more motoric modes that have been the focus of most prior work (e.g., Boria et al., 2009), but also support the more specific interactions between object and motor information predicted by the affordance-matching hypothesis. In the following, we will briefly review some important findings.

### Object manipulation knowledge guides action prediction

The affordance-matching hypothesis posits that people do not only derive manipulation knowledge for the objects relevant to their goals, but also for the objects relevant for the goals of others (for a similar argument, see Creem-Regehr et al., 2013). This knowledge directly constrains the motor behaviors expected from the other person, allowing for efficient action prediction. Indeed, there is ample evidence from studies in children and adults that human observers do not only interpret actions *post-hoc*, but actively predict how they will continue (e.g., Flanagan and Johansson, 2003; Falck-Ytter et al., 2006; Uithol and Paulus, 2013). Several studies have demonstrated that these predictions are directly informed by objects and knowledge about the movements required for their effective manipulation. Hunnius and Bekkering (2010), for example, have revealed that when children observe others interacting with objects, their gaze reflects their predictions about the actions to follow. Seeing somebody reach and grasp a cup, therefore, evokes gaze shifts towards the mouth, while seeing somebody grasp a telephone evokes gaze shifts towards the ear, providing direct evidence that an object’s typical manipulation can guide action prediction.

Studies on adults similarly support the notion that observers routinely rely on object knowledge to predict forthcoming actions. A range of studies has established that when people see somebody else next to an object, the most effective grip to interact with it is activated, as if they were in the position of the observed actor (cf. Costantini et al., 2011; Cardellicchio et al., 2013). Moreover, consistent with the affordance-matching hypothesis, the activations of these actions has a predictive function and biases perceptual expectations towards these actions. In a recent study by Jacquet et al. (2012) participants identified, in a condition of visual uncertainty, complete and incomplete object-directed actions. For each object, an optimal (low biomechanical cost) and sub-optimal (high biomechanical cost) movement was presented. As predicted from affordance matching, participants more easily identified the movements optimally suited to reach a given object, in line with the idea that extracted affordances have biased visual perception towards these actions.

Other studies confirm that contextual information about the currently relevant action goals guides attention towards relevant objects (Bach et al., 2005, 2009, 2010b; van Elk et al., 2009). Social cues—particularly another person’s gaze—are one such source of information (see Becchio et al., 2008 for a review). In human actors, gaze is typically directed at the target of an action, even before it is reached (Land and Furneaux, 1997; Land et al., 1999). Human observers, as well as some primates, are aware of this relationship and exploit it to predict the action’s target (Phillips et al., 1992; Call and Tomasello, 1998; Santos and Hauser, 1999; Scerif et al., 2004). If this is the case, then other people’s gaze should determine for which objects manipulation knowledge is retrieved. Indeed, Castiello (2003; see also Pierno et al., 2006) reported that observing object-directed gaze primes reaches towards the object, just as if one were directly observing this action. Similarly, research using fMRI has shown that observing an object-directed gaze activates similar premotor and parietal regions as when actually observing an action towards this object (Pierno et al., 2006, 2008). These findings directly support our contention that gaze implies a goal to interact with an object, which in turn activates the necessary actions (cf. “intentional imposition”, Becchio et al., 2008).

Another important source of information is the other objects in a scene, which—if they complement the object the actor is wielding—can directly suggest an action goal (e.g., a key and a keyhole suggest the goal of locking/unlocking a door, but key and a slot of a screw do not). It has been known for a while that patients with visual extinction, who are generally unable to perceive more than one object at a time, are able to perceive two objects if the objects show such a functional match (Riddoch et al., 2003). Importantly, perception was further enhanced when the spatial relationship between the objects matched the objects’ required manipulation (e.g., corkscrew above rather than below a wine bottle), supporting the idea that implied goals suggested by functionally matching objects drove the retrieval of manipulation knowledge (for a similar effect in healthy adults using the attentional blink paradigm, see McNair and Harris, 2013).

In a behavioral study, we directly tested the idea that action goals implied by potential action recipients are enough to activate the required manipulation (Bach et al., 2005). Participants had to judge whether a tool (e.g., a credit card) was handled correctly according to its typical manipulation, but varied whether a recipient object was present that either matched the typical function of the object or did not (e.g., slot of a cash machine, or a slot of ticket canceller), while controlling whether the action could be physically carried out (i.e., the credit card could just as easily be inserted into the slot of the ticket canceller as into the cash machine). As predicted, we found that manipulation judgments of others’ actions were sped up by the presence of functionally congruent objects, in line with the idea that implied action goals pre-activate associated manipulations (for similar findings, see van Elk et al., 2009; Yoon et al., 2010; Kalénine et al., 2013).

### Observed manipulations confirm action interpretations

The above studies show that affordances of objects combine with contextual and social information about the actor’s goals in the prediction of forthcoming actions. What happens if such a prediction is indeed confirmed? According to the affordance-matching hypothesis, each function of an object is associated with a specific manipulation that is necessary to achieve this goal. A match between an actually observed action and this predicted manipulation allows observers to infer the action’s function: the object can lend the action its meaning.

On a general level, this predicts that, next to movements, objects should be a prime determinant of how actions are understood and distinguished from one another. From the developmental literature, such object-based effects of action understanding are well known. In a seminal study, Woodward (1998) habituated infants to seeing another person reach for one of two objects. After habituation, the position of the objects was switched, so that the same movement would now reach a different object, and a different movement would reach the same object. The results showed that, indeed, infants dis-habituated more to changes of the objects than to changes of the movements, even though the change of movement was more visually different from the habituated action. This suggests that infants interpret other people’s reaches as attempts to reach a particular object, such that changes of these objects, but not of the movements required to reach them, change the “meaning” of the action. Indeed, the effects were absent when the object was grasped by an inanimate object with similar shape as the human arm, suggesting that the effect indeed relates to the goals associated with the objects (but see Uithol and Paulus, 2013, for a different interpretation). Moreover, other studies show that the effects depend on the infants’ prior interaction experience with the objects, in line with the idea that the effects emerge from ones’ own object knowledge (Sommerville et al., 2005, 2008).

Of course, this study only shows on a basic level that objects determine the inferred goal of an observed motor act. Since then, it has been demonstrated that these goal attributions indeed rely on a sophisticated matching of observed actions to the manipulations required to interact with the target object. For example, in the case of simple grasps, the volumetrics of the objects provide affordances for a specific type of grip, with larger objects affording whole hand power grips and smaller objects affording precision grips (e.g., Tucker and Ellis, 1998, 2001). There are now several studies—in children and adults—that show that inferences about a reach’s goal are based on such grip-object matches. For example, Fischer et al. (2008) demonstrated that simply showing a certain type of grip triggers anticipative eye movements towards a goal object with a corresponding shape, implying an identification of the action goal based on affordance matching. This capacity is well established already in infants. Daum et al. (2009) have shown that at 6–9 months, children routinely establish such relationships between grasps and goal objects, showing dis-habituation when grasping an object that was incongruent with the initial grip. Even at this age, therefore, children “know” that different objects require different grips, and they can anticipate the goal of an action based on the matching between this affordance, and the observed grip.

Importantly, and in line with the affordance-matching hypothesis, these effects are guided by the same object manipulation knowledge that guides an individual’s own actions. Infants’ ability for affordance matching directly depends on their ability to exploit these affordances for their own actions. Only those children who used accurate pre-shaping of their own hand to the different object types used this match information to anticipate which object would be grasped (Daum et al., 2011). Similar evidence comes from a study tracking infants’ eye movements. As in adults, congruent shapes of the hands allowed infants to anticipate (fixate) the goal object of a reach, and this ability was dependent on their own grasping ability (Ambrosini et al., 2013).

Such effects are not restricted to grasping. In tool use, the manipulations one has to perform with a given tool to realize its function are, if anything, even more distinct (e.g., the swinging motion of hammering, the repetitive finger contractions when cutting with scissors). In an early study, we therefore asked whether a tool that was applied appropriately to a goal object would help identify the goal of an action (Bach et al., 2005). Participants had to judge whether two objects could, in principle, be used together to achieve an action goal (e.g., screwdriver and slot screw vs. screwdriver and slot of a keyhole), but had to ignore whether the orientation of the tool relative to the goal object matched the associated manipulation (e.g., same orientation for screwdriver and screw, but orthogonal orientations of scissors and piece of paper). We found that incongruent manipulations slowed down judgment times, but only for object combinations that suggested a goal; for those that did not, even when otherwise physically possible (e.g., a screwdriver that would fit into a keyhole), this effect was completely eliminated. This is therefore in line with the idea that goal inferences are automatically verified by matching the actually observed action with the required manipulation, but if no potential goal is identified in the first place, such a matching does not take place. Similar findings have been provided by different labs in both adults (van Elk et al., 2009) and children (Sommerville et al., 2008).

If this conception of action understanding is correct, one would predict that object information is key to the comprehension of observed actions, and should therefore also involve strongly overlapping brain regions. We have recently tested the idea that object-related activation is the primary driver of action understanding (Nicholson et al., submitted). In an fMRI study we showed participants a sequence of different everyday actions—such as pouring a glass of wine, paying with a credit card, or making coffee—while directing their attention either towards the movements involved, the objects used or the goals of the actions. Consistent with the affordance matching hypothesis, goal and movement tasks produced markedly different brain activations, while activations in the goal and object task were—to a large extent—identical.

### Affordance matching guides imitation

Evidence that affordance matching guides action interpretation comes from research on imitation. There is ample evidence that children’s imitation does not reflect a faithful copying of the observed motor behavior, but is based on the goal. Unless the specific motor behavior appears crucial to goal achievement (or for fulfilling social expectations, Over and Carpenter, 2012), children try to achieve the same goal with actions that are most appropriate to their circumstances, that is, they *emulate* rather than *imitate* the observed action (Gergely et al., 2002; see Csibra, 2008, for review). If this is the case, and if affordance-matching contributes to these goal inferences, then we should find that actions are specifically imitated when matching the affordances of their goal object.

This indeed seems to be the case. When children observe others reach with their hand to either their ipsilateral or contralateral ear, they primarily attempt to reach for the same target object (i.e., the correct ear), but do so predominantly with an ipsilateral reach, thus ignoring how the actor achieved the goal, and choosing the most appropriate reach for themselves (Bekkering et al., 2000). As seen in Woodward’s study, therefore, the goal object determined the interpretation of the action, and this goal served as the basis for imitation while the movement form was neglected (for further discussion on the role of goals in imitation, see Csibra, 2008; see Uithol and Paulus, 2013, for a critical look at such interpretations).

Studies on adults confirm that specifically those actions are imitated, which match the affordances of the goal objects. Humans have a general tendency to automatically imitate other people’s actions (Chartrand and Bargh, 1999; Brass et al., 2000; Bach et al., 2007; Bach and Tipper, 2007). Wohlschläger and Bekkering (2002) showed that imitation of simple finger tapping movements is enhanced for the most effective movements towards the goal objects (marked spots on a table), and this effect has been linked to the inferior frontal gyrus, one of the assumed homologs of monkey area F5, where mirror neurons have first been discovered (Koski et al., 2002). In a recent study, we revealed similar effects for automatic imitation of reach trajectories. Observers specifically tend to imitate the direction of observed reaches, if the configuration of the hand matched the size of the goal object (Bach et al., 2011). Other studies have revealed similar findings, showing that muscle activation induced by transcranial magnetic stimulation (TMS) to the motor cortex when watching others grasp objects is higher when the observed grasps match the affordances of the goal object (e.g., Gangitano et al., 2004; Enticott et al., 2010).

## Relations to recent accounts of mirror neurons and action understanding

The above review shows that the affordance-matching hypothesis can unify a range of recent findings on children’s and adult action observation. However, we believe that it is also in line with the single cell evidence, particularly with findings about mirror neurons in the macaque premotor and parietal cortices (di Pellegrino et al., 1992; Fogassi et al., 2005). Recently, several theorists have started to re-evaluate the thesis that mirror neurons are part of a bottom-up mechanism for action recognition (e.g., Rizzolatti and Craighero, 2004), and—in line with the affordance matching hypothesis—instead highlighted their role in matching sensory input to top-down action expectations (e.g., Kilner et al., 2007a; Csibra, 2008; Liepelt et al., 2008a; Bach et al., 2010b, 2011).

Csibra (2008), for example, argues that initial inferences about the goal of an observed action are not based on motoric matching, but driven by contextual information in the scene (e.g., prior knowledge about others’ intentions, eye gaze, emotional expression, etc.). Once such an initial goal has been inferred, the job of the mirror neurons is to produce an “emulation” of an action that would be suitable to achieve this goal, based on the observers’ own action knowledge. Their firing signals a match between observed action and this emulation, and therefore allows observers to confirm that the correct goal was inferred. In contrast, if there is no such match between predicted and observed action, the inferred goal is revised, and a new—hopefully better matching—emulation can be produced. As proposed by affordance matching, this emulation does not only serve such an interpretative function, but also aids action prediction. Especially during visual uncertainty, the emulation can be used to “fill in” action information not obtained directly through perception (for recent evidence for such a filling in, see Avenanti et al., 2013a).

Kilner’s (2007a; see also Grafton and de C. Hamilton, 2007) predictive coding account follows a similar principle. The mirror system is seen to be part of a hierarchy of reciprocally connected layers, with goal information at the top and motor or kinematic information at the bottom levels. As in Csibra’s model, initial goal inferences are derived from contextual information in the scene. Guided by the observers’ own action knowledge, these goals are translated into predictions for forthcoming movements and fed into the lower levels. Incoming sensory stimulation is matched against this signal and elicits a prediction error in case of a mismatch. The next level up can then alter its own prediction signal to reduce this mismatch. As in (Csibra’s 2008) model, this sparks a chain of forward and backward projections through the interacting levels, where different goals can be “tried out”, until emulation and visual input overlap and the prediction error is minimal.

In such views, therefore, the firing of mirror neurons is interpreted not as the autonomous detection of an action goal (Rizzolatti and Craighero, 2004), but as the detection of a predicted motor act that is in line with a previously inferred action goal (cf. Bach et al., 2005, 2010b). The affordance-matching hypothesis agrees with these general ideas. Both of these prior views, however, are relatively vague about how contextual information influences prediction and interpretation. With the notion of coupled function and manipulation knowledge, the affordance-matching hypothesis introduces a specific mechanism via which such goal inferences can be made and translated into predictions of forthcoming motor acts. Indeed, in the following we will review some key pieces of evidence that suggest that response conditions of mirror neurons are not only in line with predictive accounts (see Kilner et al., 2007a; Csibra, 2008), but specifically with the notion that knowledge of how to manipulate objects drives these prediction processes.

### Mirror neurons and affordance matching

A classical finding is that mirror neurons fire only for actions that are directed at an object (be it physical, such as a peanut, or biological, such as a mouth), but not if the same body movement is observed in the absence of an object (i.e., mimed actions). This finding is often interpreted as showing that mirror neurons encode the goal of an action: the goal of *reaching* *for something* rather than the motor characteristics of the reaching act itself (Umilta et al., 2001; Rizzolatti and Craighero, 2004). However, in the light of the affordance matching hypothesis, an alternative interpretation is that the firing of the mirror neurons confirms a specific action that has been previously predicted, based on the affordances of the object (e.g., a reach path on track towards the object location with a grip that is appropriate for the object size). In the absence of an object, no specific grasp is predicted, and hence the mirror neuron remains silent even if one occurs (for a similar argument, see Csibra, 2008). Such an interpretation does not deny that the firing of mirror neurons is goal-related; however, rather than encoding the abstract goal of grasping itself, it suggests that the firing of mirror neurons might signal a movement that matches a functional object manipulation.

Another important aspect are the various reports of object specificity of mirror neuron responses. Consider, for example, that mirror neurons fire consistently only for motivationally relevant objects, like food items (Gallese et al., 1996; Caggiano et al., 2012). For abstract objects, such as spheres or cubes, firing subsides quickly after the initial presentations. This is directly in line with our proposal that the selection of objects for which the affordances are extracted is guided by the functional relevance of the objects towards the actor’s goals. Consistent with this interpretation, it has recently been revealed that while a large number of mirror neurons respond preferentially to objects that had been previously associated with reward, a smaller number fire specifically for objects that are not associated with reward (Caggiano et al., 2012). This separate encoding of the same motor acts towards different object types reveals that mirror neuron responses are dependent on object function: they allow observers to disambiguate predicted action goals (here: to gain a reward or not) by matching them to the different movements suitable to achieve these goals.

Another important finding is that mirror neurons in the parietal cortex fire based not on the observed movement itself, but based on its ultimate goal (Fogassi et al., 2005). That is, even when merely observed, the same reaching action is encoded by different mirror neurons depending on whether it is performed with the ultimate goal of placing the objects somewhere else, or eating it. Again, this finding is often interpreted as revealing a coding of the action goal, but it is also in line with the matching of different predictions based on object context. The reason is that, in this experiment, the different goals were not extracted from movement information (the initial grasps were identical for both goals), but by object information: grasps to place were signaled by the presence of a suitable container in reach of the model, while grasps to eat were signaled by the absence of this container (see supplementary material, Fogassi et al., 2005). The finding therefore provides direct support for affordance-matching: mirror neurons fire not because they autonomously derive the goal of the action, but because they detect an action that has been predicted from the presence of objects (for a similar argument, see Csibra, 2008).

An untested prediction of the affordance-matching hypothesis is that mirror neurons should encode the specific motor act expected by the object. They should therefore fire specifically, or most strongly, for a motor act afforded by the object. A mirror neuron encoding precision grips during own action execution should fire most strongly not only if a precision grip is observed, but also if the observed object is one that affords a precision grip (i.e., a small object). In contrast, a mirror neuron encoding whole hand prehension should fire most strongly if a power grip is observed towards an object that affords a power grip (a large object). Some suggestive evidence for such an object-action matching process was provided by Gallese et al. (1996). They reported, first, that some grasping and manipulation-related mirror neurons only fired for objects of specific sizes, but not for larger or smaller objects (but without providing details on whether size and grip had to match). Second, they reported that mirror neurons do not fire even if the monkey sees a grasp and an object, unless the hand’s path is indeed directed towards the object, revealing mirror neuron responses do not only require object presence, but (1) a specific type of object; and (2) a precise targeting of the action towards the object, in line with a matching of action to object affordances.

Similar evidence comes from recent studies on humans that have linked the matching of observed movements to those afforded by the objects to areas in premotor and parietal cortex, the brain regions where mirror neurons have been discovered in the macaque monkey. During grasp observation, these regions become activated when computing the match between grips and objects (Vingerhoets et al., 2013), and respond more strongly for reach errors, specifically when a reach deviates from the path predicted by the object (Malfait et al., 2010). Similarly, in the domain of tool use, they are involved in computing the match between an observed manipulation and the manipulation required to realize the object’s function (Bach et al., 2010b). Of course, the conclusion that these affordance-matching related activations in humans indeed reflect mirror neurons need to be interpreted with caution, as none of these studies assessed a role of these regions in motor performance. However, it is noteworthy that the response of these regions correlates with the observer’s sensorimotor experience with the actions (Bach et al., 2010b), a criterion that has been proposed for identifying mirror neurons in humans (cf. Calvo-Merino et al., 2005, 2006). Moreover, the parietal activations overlap tightly with the foci identified in a recent meta-analysis on grasp execution (Konen et al., 2013), and the peak coordinates overlap with regions with mirror properties identified by a recent meta-analysis (Molenberghs et al., 2012). Activations in the premotor cortex are particularly close, with peak voxels in the Malfait et al. (2010) and our own study (Bach et al., 2010b) falling within 5 mm of the peaks identified in the meta-analysis.

### Open questions and further predictions

An open question is how these affordances, which ultimately inform mirror neuron responses, are derived. During own action execution, this role appears to be played by the canonical neurons, which fire both when the monkey executes a specific grip and when it views an object that can be manipulated with this grip. These neurons therefore appear to derive object affordances and specify how an object should be interacted with. Indeed, if the bank region of F5—the region where canonical neurons are primarily located—is inactivated, object-directed grasping is disrupted as well (Fogassi et al., 2001). In contrast, inactivation of the convexity of F5, the area where mirror neurons are primarily located, does not produce such execution impairments, merely slowing down the monkey’s actions. It has therefore been argued that, while canonical neurons derive the appropriate grip, mirror neurons play a monitoring role, providing the monkey with “assurance” (p. 583) that its action is on track (Fogassi et al., 2001, see also, Bonaiuto and Arbib, 2010; Fadiga et al., 2013).

A similar division of labor—between deriving object affordances and matching the actually observed action towards this prediction—might happen during action observation. A recent study (Bonini et al., 2014) has provided evidence for specialized “canonical-mirror neurons” in monkey area F5 that appear to play the role of affordance extraction for other people’s actions. These neurons respond both when the monkey sees an object in extrapersonal space, and if somebody else performs an action towards it. In contrast to typical canonical neurons, their responses are not constrained to the monkey’s peripersonal space, and to an object orientation most suitable for grasping. In line with our hypothesis, the authors therefore argued that these neurons might provide a “predictive representation of the impending action of the observed agent” (p. 4118).

Other data points may, at first glance, show a less obvious link to the affordance-matching hypothesis. One example is the finding that a subset of mirror neurons that respond to grasping will also respond—after training—to grasps of the actions with a tool (Umilta et al., 2001; Ferrari et al., 2005). This finding is often taken as evidence that mirror neurons encode higher-level goals (“grasping”) rather than the relevant motor behaviors themselves. A slightly different explanation is provided by the affordance-matching hypothesis. On this view, mirror neurons do not generalize across different motor acts subserving the same goals, but across different perceptual cues that are informative of action success. For example, a mirror neuron that originally tests grasp success by monitoring fingers closing around an object may learn that the same success condition is met when the end of pliers close around the object. In other words, learning enables the tool tips to be treated like the tips of one’s own fingers (cf. Iriki et al., 1996). Such an interpretation is not inconsistent with the encoding of goals of mirror neurons. However, rather than encoding the abstract goal of grasping something, mirror neurons would encode a lower-level perceptual goal state of effectors—be they part of a body or of a tool—close around a target object.

A similar argument can be made for the finding that a large number of mirror neurons are only *broadly congruent*, typically showing a more specific tuning during action execution than observation. For example, during execution, one neuron might fire only when the monkey grasps an object with its hand, while during observation it may fire for grasps with both hands and mouth (cf. Gallese et al., 1996). If one takes a monitoring view of mirror neurons, such differences may emerge naturally from the differential availability of perceptual cues during action and perception. Note for example that during observation one has a view of other people’s hands and mouths, but not during one’s own actions. A neuron that simply checks whether a body part is on a path towards a target object can therefore perform this test on hands and mouths during perception, but only for hands during execution, giving the impression of a stricter tuning. We believe that such and other differences in the input available during perception and action—such as prior action selection processes or different action capabilities of monkey and model—might give rise to the otherwise surprising response profiles of broadly congruent mirror neurons. However, to what extent such hypotheses can be supported by evidence is currently unclear, and a full integration into the current model will therefore be the subject of future work.

## Extension to other action types

The affordance-matching hypothesis contrasts with initial views of action understanding as a bottom-up process (e.g., Rizzolatti et al., 2001; Rizzolatti and Craighero, 2004; Iacoboni, 2009), where observers simulate the outcome of actions based on their prior knowledge about motor commands and their perceptual consequences. Our contention is not that affordance matching is the only way that object-directed actions can be understood, but that it provides a fast and efficient means for action interpretation and prediction for well-known everyday object directed actions. Actions involving unknown objects, for example—or actions with common objects used in unusual ways—might benefit from a bottom up approach that combines a simulation of the motor actions with the mechanical properties of the objects to derive likely action outcomes. Indeed, recent work has revealed such processes of “technical reasoning” during planning of object-directed actions (e.g., Osiurak et al., 2009), and studies on action observation have shown that mirror-related brain areas become activated specifically for actions that are not known (e.g., Bach et al., 2011, 2014; Liew et al., 2013). However, even in these cases top-down processes can contribute, if one assumes that the relevant function and manipulation knowledge is tied not only to objects as a whole, but to certain object characteristics as well (e.g., any hard object can be used for hammering if it is brought down in force onto the recipient object). Future work will need to establish more closely the boundary conditions that decide which of these two pathways to action understanding and prediction are chosen.

Our discussion has so far focused on manual object-directed actions, which are often seen as the paradigmatic case of human action. However, there is no reason why similar processes may not govern the perception for actions made with other body parts. Walking, for example, one of our most frequent daily actions, happens in an object context, and the paths we take are governed by the objects (and people) surrounding us, and their relevance to our goals. Such actions should therefore be predicted and interpreted in a similar manner as manual actions. Thus, in the same way as observers can predict that a thirsty actor will grasp a glass of water in front of them, they can predict the path the actor would take to a glass on the other side of a room.

The same argument can be made for other cues that guide our social interactions, such as eye gaze and the emotional expressions that typically accompany it. Most of these actions are again object-directed, and observers implicitly understand this object-directedness (Bayliss et al., 2007; for a review, see Frischen et al., 2007; Wiese et al., 2013). People look at objects and may smile or frown in response to them. Knowing how objects relate to the actor’s goals therefore allows one to predict future looking behavior and emotional expressions, which, in turn, can confirm these goal inferences. Various studies now confirm the presence of prediction or top down effects in gaze and expression understanding. For example, Wiese et al. (2012) recently demonstrated that the classical gaze cuing effects—the extent to which an observer’s attention follows another person’s gaze—is not driven only by stimulus information but by intentions attributed to the other person.

For other types of action, the link to object knowledge is less clear. Sometimes, observers do not have any information about objects used in an action, for example because the relevant objects are hidden from view (e.g., Beauchamp et al., 2003), or because the action is pantomimed (e.g., during gesturing, Hostetter and Alibali, 2008; Bach et al., 2010a). Here, therefore, the required manipulation cannot be retrieved from the visible objects, but from a much larger variety of possible manipulations in memory. Identifying an object that would match this movement should therefore be relatively slow and effortful, unless the observed movements are highly idiosyncratic, or likely objects have already been pre-activated by assumptions about the actor’s goals or contextual cues. However, as soon as a matching object-manipulation pairing is identified, the action can be interpreted and predicted in a similar manner as for fully visible actions (for evidence for such a prediction of pantomimed actions, see Avenanti et al., 2013b, albeit without linkage to object centered mechanisms).

Intransitive actions—such as stretching or spontaneous smiles—are another example. They produce motor activation just like the observation of object directed actions (Costantini et al., 2005; Romani et al., 2005; Urgesi et al., 2006), but they are, by definition, excluded from the present model. As they are neither directed at an object, nor do they involve objects as an instrument, object knowledge can therefore not contribute to their interpretation and prediction. We speculate, however, that their processing may follow similar principles. As it is the case for object-directed actions, intransitive actions link certain kinds of movement (e.g., stretching) with a specific function, typically with reference to one’s internal state (e.g., to relieve some symptoms of tiredness). If such a linkage exists, it can provide similar predictive and interpretative functions as the analogous knowledge about objects. Knowing about someone’s internal state, may allow one to predict forthcoming actions. Observing these actions, in turn, can then disambiguate possible interpretations about the individual’s internal states. However, there is still considerable debate in the literature about how intransitive actions are processed when observed. Future research needs to disentangle these processes, and more closely describe how they interact with one’s (inferred) knowledge about a person’s internal states.

## Conclusions

Several recent proposals have challenged the idea that a motoric matching process, instantiated by the mirror neuron system, is the key driver of action understanding in humans. Yet, they have left open which alternative source of information could be used instead. The affordance-matching hypothesis posits a key role of objects. It specifies how action prediction and interpretation arises from a combination of object knowledge—how it is used and what it is for—and the actor’s current goals and motor behaviors. Such a view can account for a variety of findings and integrates them into a common framework. Moreover, it provides an intuitive account of how the understanding of others’ actions can be grounded in one’s own experiences. For the perception of everyday object-directed actions, this grounding does not result from a matching of motor parameters, but is based on the identity of the objects, and one’s prior experiences about their function and use.

## Conflict of interest statement

The authors declare that the research was conducted in the absence of any commercial or financial relationships that could be construed as a potential conflict of interest.
